# Study protocol for a comparative effectiveness trial of two models of perinatal integrated psychosocial assessment: the PIPA project

**DOI:** 10.1186/s12884-017-1354-0

**Published:** 2017-07-20

**Authors:** Nicole Reilly, Emma Black, Georgina M. Chambers, Virginia Schmied, Stephen Matthey, Josephine Farrell, Dawn Kingston, Andrew Bisits, Marie-Paule Austin

**Affiliations:** 1 Perinatal & Women’s Mental Health Unit, St John of God Health Care & University of New South Wales, St John of God Burwood Hospital, 13 Grantham St, Burwood, NSW 2134 Australia; 2 National Perinatal Epidemiology and Statistics Unit, Centre for Big Data Research in Health and School of Women’s and Children’s Health, University of New South Wales, Level 1, AGSM Building (G27), Sydney, NSW 2052 Australia; 3 School of Nursing and Midwifery, Western Sydney University, Building EBLG Room 33, Parramatta South Campus, Parramatta, NSW 2150 Australia; 4 School of Psychology, University of Sydney & Infant, Child & Adolescent Mental Health Service, South Western Sydney Local Health Network, Mental Health Centre (Level 1: ICAMHS), Locked Bag 7103, Liverpool BC, NSW 1871 Australia; 5 Faculty of Nursing, University of Calgary, 2500 University Drive, Calgary, AB T2N 1N4 Canada; 6 Royal Hospital for Women, Barker St, Randwick, NSW 2031 Australia

**Keywords:** Depression screening, Psychosocial assessment, Antenatal, Maternal mental health, Clinical effectiveness, Cost effectiveness

## Abstract

**Background:**

Studies examining psychosocial and depression assessment programs in maternity settings have not adequately considered the context in which psychosocial assessment occurs or how broader components of integrated care, including clinician decision-making aids, may optimise program delivery and its cost-effectiveness. There is also limited evidence relating to the diagnostic accuracy of symptom-based screening measures used in this context. The Perinatal Integrated Psychosocial Assessment (PIPA) Project was developed to address these knowledge gaps. The primary aims of the PIPA Project are to examine the clinical- and cost-effectiveness of two alternative models of integrated psychosocial care during pregnancy: ‘care as usual’ (the SAFE START model) and an alternative model (the PIPA model). The acceptability and perceived benefit of each model of care from the perspective of both pregnant women and their healthcare providers will also be assessed. Our secondary aim is to examine the psychometric properties of a number of symptom-based screening tools for depression and anxiety when used in pregnancy.

**Methods:**

This is a comparative-effectiveness study comparing ‘care as usual’ to an alternative model sequentially over two 12-month periods. Data will be collected from women at Time 1 (initial antenatal psychosocial assessment), Time 2 (2-weeks after Time 1) and from clinicians at Time 3 for each condition. Primary aims will be evaluated using a between-groups design, and the secondary aim using a within group design.

**Discussion:**

The PIPA Project will provide evidence relating to the clinical- and cost- effectiveness of psychosocial assessment integrated with electronic clinician decision making prompts, and referral options that are tailored to the woman’s psychosocial risk, in the maternity care setting. It will also address research recommendations from the Australian (2011) and NICE (2015) Clinical Practice Guidelines.

****Trial Registration**:**

ACTRN12617000932369

## Background

One in five women experience some form of mental health morbidity during pregnancy and the first postnatal year (the perinatal period). A large meta-analysis of 28 studies reported that up to 12.7% and 21.9% of women will experience major depression during pregnancy and in the first twelve months postpartum [1], respectively, while up to 21% of women meet diagnostic criteria for at least one anxiety disorder during pregnancy [2, 3]. Recent evidence from birth cohorts in France and Australia also suggest that up to 40% of women with untreated prenatal depression continue to experience symptoms four and five years postpartum [4, 5].

The health service systems in place for routine maternity care in a number of countries have provided a unique opportunity to introduce perinatal mental health promotion, prevention and early intervention programs. In Australia, routine psychosocial assessment – a broad clinical evaluation of a woman which aims to help facilitate the provision of comprehensive, quality clinical care – is central to many of these programs. In the perinatal mental health context, such an approach encompasses enquiry into a range of risk and protective factors known to affect perinatal mental health for both mother and infant, including her current and past psychological health and social circumstances. This assessment may be enhanced by the use of relevant ‘screening’ or case detection tools, such as the Edinburgh Postnatal Depression Scale (EPDS) which asks about symptoms of depression occurring in the previous seven days [6]. The aim of this assessment is to identify the presence of current symptoms or psychosocial factors known to be associated with poorer maternal mental health so that appropriate care can be provided and outcomes for women improved [7].

In the last decade Australia has become a world leader in the development of national policy and clinical practice for perinatal mental health. Examples of key Australian national initiatives that are inclusive of routine depression screening and psychosocial assessment include the *beyondblue* National Postnatal Depression Program (2001–2005) [8], the *beyondblue* National Action Plan for Perinatal Mental Health (2008) [9] and the National Perinatal Depression Initiative (NPDI; 2008–2015), which also supported the development of Australia’s first national Clinical Practice Guidelines for Depression and Related Disorders in the Perinatal Period [7]. The need for such screening and assessment programs to be well integrated with clinical guidance for appropriate care planning, and across health care disciplines and sectors, has been highlighted [10–12].

In Sydney, where the PIPA Project is based, the SAFE START perinatal mental health policy directive and clinical practice guidelines have been in place at a number of large public maternity hospitals since 2010 [13–15], with the Integrated Perinatal Care (IPC) model of care implemented in some metropolitan settings prior to this time [16]. The SAFE START policy and guidelines specify a model of perinatal psychosocial assessment that includes psychosocial assessment at the first antenatal hospital appointment (the ‘booking-in’ visit), nominates a woman’s psychosocial ‘risk level’ and recommends further clinical action where appropriate. For women with higher levels of risk, clinical review at a multidisciplinary case discussion (MCD) meeting is required [13–15].

An evaluation of the SAFE START model of care noted a number of limitations, including an overrepresentation of ‘false positive’ women discussed at MCD meetings. In other words, women some were being referred to the MCD, when these women either did not require further assessment or referral, or could have instead been further monitored by their midwives, without discussion at MCD [17]. One potential reason for this high rate of referrals to the MCD meetings is the lower threshold set by SAFE START for further referral, which may result in inadvertent over-servicing, reduced system efficacy, increased costs through service duplication, and increased anxiety for women being incorrectly identified as ‘at risk’ [18].

In response to these limitations in the SAFE START model, an alternative approach – the Perinatal Integrated Psychosocial Assessment (PIPA) model – was developed by Austin and colleagues and will be compared with SAFE START (care as usual) in the current study.

## Methods

### Clinical context and study definitions

**Fig. 1:**
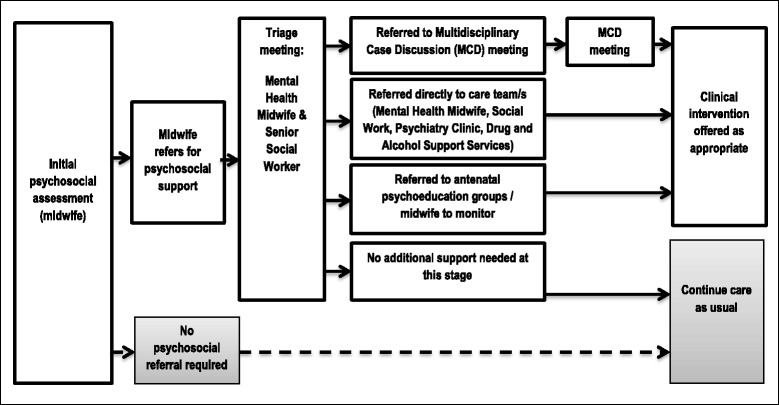
Psychosocial care pathway

At the initial antenatal booking in appointment, midwives at the participating site decide whether each woman in their care requires a psychosocial referral based on their responses to the routine psychosocial assessment. Once a woman has been flagged for psychosocial referral, the mental health midwife and senior social worker allocate women to the relevant service/s at a weekly ‘triage’ meeting, according to their psychosocial presentation. Referral can be made to one or more on-site components of the maternity psychosocial service, including the multidisciplinary case discussion (MCD) meeting, psychiatry clinic, mental health midwife, social work, drug and alcohol support services and antenatal psycho educational and therapy groups (see Fig. 1).

For the purposes of the PIPA Project, the following definitions will apply:

*Referral:* will be defined as the initial referral made by the midwife to one or more of the on-site support options. *NOTE: Referrals to services external to the participating site are outside the scope of data collection for the current project, as are referrals made at later antenatal appointments (*i.e.*, not the initial booking-in appointment).*

*‘Correct’ referral:* a clinically appropriate referral made by the midwife, as determined by the consensus opinion of senior psychosocial clinicians at the weekly ‘triage’ and MCD meetings.

*‘Incorrect’ referral:* a clinically inappropriate referral made by the midwife, as determined by the consensus opinion of senior psychosocial clinicians at the weekly ‘triage’ and MCD meetings.

### Aims

The PIPA Project will address three primary aims and one secondary aim, as follows:

*Primary Aim 1: Clinical effectiveness outcomes:* To compare the two models of care in relation to: i) the proportion of women identified as ‘at risk’ and referred by the midwife conducting the initial psychosocial assessment to the various referral pathways (e.g., mental health midwife, social work, MCD meeting), stratified by comparable levels of risk in each model of care (see Table 1); and ii) the proportion of ‘correct’ and ‘incorrect’ referrals from the initial psychosocial assessment in each model.

**Table 1:** Comparison of key features of the SAFE START and PIPA models of integrated psychosocial assessment

	Model of integrated psychosocial assessment	
	SAFE START model (Care as usual)	PIPA model (Alternative model)
Psychosocial assessment measures	EPDS, SAFE START psychosocial questions	EPDS, ANRQ-R (psychosocial questions); clinician concerns
Psychosocial risk levels	*Three levels of psychosocial risk, defined as:* *Level 1*: no specific vulnerabilities or risk *Level 2*: *one or more risk factors* of variable severity and significance including, but not limited to, low supports, multiple birth, financial stress, isolation, ‘mild-moderate’ depression or anxiety, history of mental health problem, young age. *Level 3*: *one or more of four complex risk factors* (domestic violence, involvement with child protection services, substance misuse, severe mental illness)	*Six levels of psychosocial risk, defined as:* *No risk*: ANRQ-R = 0; EPDS < 13 (Q10 = 0); no clinician concerns. *No risk on ANRQ-R* (ANRQ-R = 0) *but clinician concerns and/or EPDS = 13 or 14.* *Low risk*: ANRQ-R = 1–24 (excluding e,g., significant mental health history^a^) and EPDS < 15 (Q10 = 0). *Medium risk*: ANRQ-R = 1–24 (including e,g., significant mental health history^a^) and EPDS < 15 (Q10 = 0). *Medium-high risk*: ANRQ-R ≥25 (excluding any ‘complex’ risk factors^b^) or combination of ‘social’ risk factors^c^ or EPDS ≥15 (Q10 = 0) or childhood trauma and neglect. *High risk*: ANRQ-R >25 and other ‘social’ risk factors^c^ or any ‘complex’ risk factor (s)^b^ or EPDS Q10 ≥ 1.
MCD meeting referral threshold	Levels 2 and 3	High risk

*MCD* multidisciplinary case discussion meeting, *ANRQ-R* Antenatal Risk Questionnaire-Revised, *EPDS* Edinburgh Postnatal Depression Scale

^a^‘significant’ mental health history: involving professional help and/or had functional impact

^b^‘Complex’ risk factors : homelessness or housing instability; domestic violence; substance misuse; contact with child protection services

^c^‘Social’ risk factors: young maternal age (less than 20 years); no partner; booking-in appointment at >20 weeks gestation

**Table 2:** Data collection schedule and measures

Measure	Time 1 *All women (routinely collected data)*	Time 2 *Consenting women only*	Time 3 *Health professionals only*
Demographic information^a^	X		
EPDS^a^	X	X	
Psychosocial assessment^a^	X		
Routine referral information^b,c^	X		
Feedback survey (women)^d^		X	
Costing data	X		X
Feedback survey; observational data; key informant interviews; focus groups (health professionals)^b,c,e^			X
Whooley questions^f^		X	
GAD-2^f,g^		X	
MGMQ^f,g^		X	
MINI^f,g^		X	
Interval question^f,g^		X	
Brief help seeking items^f,g^		X	

*EPDS* Edinburgh Perinatal Depression Scale, *GAD-2* Generalized Anxiety Disorder Scale-2items, *MGMQ* Matthey Generic Mood Questionnaire, *MINI* Mini International Neuropsychiatric Interview (v6.0; mood and anxiety disorder modules only; Data will be used to address: ^a^All aims; ^b^Primary Aim 1 (clinical effectiveness); ^c^Primary Aim 2 (cost effectiveness); ^d^Primary Aim 3 (pregnant women perspectives); ^e^Primary Aim 3 (health care provider perspectives); ^f^Secondary Aim (psychometric evaluation). ^g^Completed by women allocated to the PIPA model of care only

*Primary Aim 2: Cost-effectiveness outcomes:* To quantify: i) the cost per ‘correct’ and ‘incorrect’ referral made at the initial psychosocial assessment, for each model of care; ii) the incremental cost per ‘correct’ referral made and the incremental cost per ‘incorrect’ referral averted by the PIPA model compared to the SAFE START model; and, iii) the budgetary implications of implementing the PIPA model at the participating site, based on number of women assessed.

*Primary Aim 3: Consumer perspectives:* To compare the acceptability and perceived benefit of each model of care from the perspective of both pregnant women and their healthcare providers.

*Secondary Aim: Psychometric properties of screening tools:* To examine the sensitivity, specificity, positive and negative predictive values, and positive and negative likelihood ratios of the EPDS [6], ‘Whooley’ depression questions [19, 20], the Generalized Anxiety Disorder Scale [GAD-7] and its short form [GAD-2] [21], and Matthey Generic Mood Questionnaire (MGMQ) [22] when used during pregnancy, using the Mini International Neuropsychiatric Interview v6.0 [23] (MINI) as the gold standard.

### Hypotheses

We hypothesize that the PIPA model of care will be at least 15% more clinically effective, and will be more economically efficient and acceptable, than ‘care as usual’. Secondly, we expect that each of the screening measures will have acceptable psychometric characteristics when used in this population of pregnant women.

### Design

**Fig. 2:**
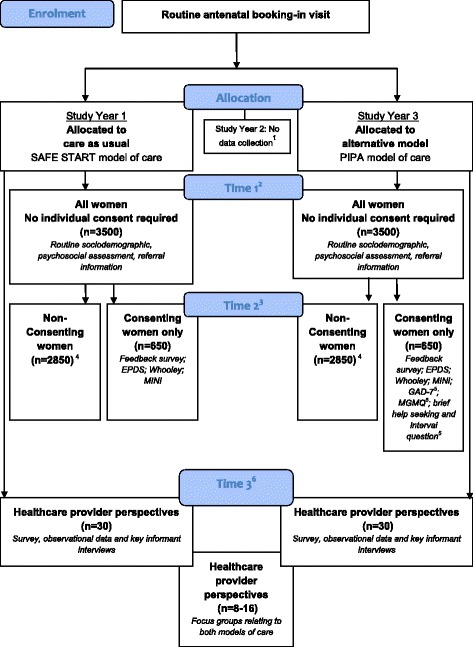
Flow chart for the PIPA Project. ^1^ No data collection in Study Year 2 due to state-level upgrade of administrative data platform at the participating site; Training in the PIPA model will take place in the final month of Year 2; ^2^ Time 1 = approx. 12–16 weeks’ gestation; ^3^ Time 2:=2 weeks post-Time 1; ^4^ Excluded from Time 2 if: declined study Expression of Interest (EoI); EoI not asked at booking-in visit; declined participation when contacted at time of Time 2; did not meet inclusion criteria at Time 2; not contacted at Time 2 due to staffing/resource limitations; ^5^ Completed by women in Group 2 only; ^6^ To examine healthcare provider perspectives, survey, observational and key informant interview data will be collected during the final 6 months of the allocation periods for Group 1 and Group 2. Focus groups will be held approx. 3-months after the implementation of the PIPA model of care

This is a comparative-effectiveness study which will compare ‘care as usual’ (the SAFE START model) to an alternative model (the PIPA model) sequentially over a 36 month period (see Fig. 2). It will employ a mixed methods design. The primary aims will be evaluated using a between-groups design, comparing the two models of care on a range of outcomes. The secondary aims will be evaluated using a within group design.

### Psychosocial assessment models of care

A summary of the key features of the SAFE START and PIPA models of care are presented in Table 1.

#### SAFE START model (care as usual)

This model of care recommends that all women complete the EPDS and SAFE START psychosocial questions at the initial antenatal booking-in appointment [13]. A paper-based version of the EPDS is completed by the woman and the total score and score on question 10 are manually scored by the midwife and recorded on a state-wide administrative database (ObstetriX; to be superseded by eMaternity), whereas the psychosocial questions are administered face-face by the midwife and responses electronically recorded in the database. The psychosocial questions cover seven key domains of risk: (1) (lack of) social supports; (2) recent stressors in the previous 12 months; (3) anxious personality style or low self-esteem; (4) history of mental health problems; (5) history of childhood abuse; (6) contact with child protective services or having a child living in the care of another person; and (7) current or recent domestic violence. Based on responses to these questions and the EPDS, women fall into one of three SAFE START psychosocial risk levels: Level 1 (no specific vulnerabilities or risk); Level 2 (endorsing one or more of a broad range of risk factors of variable severity and significance) or Level 3 (endorsing one or more complex risk factors, where ‘complex’ risk implies a high degree of psychosocial morbidity associated with greater risk of mental health disorder, lack of engagement with services and risk of poor parenting and infant outcomes, including risk of harm to the infant).

#### Perinatal Integrated Psychosocial Assessment (PIPA) model

The PIPA model of care comprises three key electronic elements:

1. Administration of the Antenatal Risk Questionnaire-Revised (ANRQ-R) questionnaire (covering a range of known psychosocial risk factors) and the EPDS (including four additional questions to aid more guided exploration of recent thoughts of self-harm, among women who endorse EPDS question 10) by the midwife, with responses recorded in eMaternity;
2. A computer-based clinician decision-support algorithm, which generates the woman’s psychosocial risk profile and self-harm risk scores (based on the ANRQ-R and EPDS), summarises the presence/combinations of identified risk factors and articulates six psychosocial risk levels, ranging from No Risk to High Risk;
3. Immediate referral prompts for the midwife conducting the assessment. These clinician prompts are embedded within eMaternity, and are tailored to the woman’s psychosocial risk profile and the on-site services available.

The ANRQ-R is a revised version of the validated ANRQ [24], incorporating the following psychosocial risk domains: possible mental health history (identifying *significant* episodes in terms of impact on function and/or seeking treatment); history of physical, sexual or emotional abuse; current/recent intimate partner violence; level of practical supports; quality of relationship with mother (in childhood) and current partner (if partnered); anxious or perfectionistic personality style; current or recent substance use; and significant levels of stress in the last 12 months as well as questions relating to (homelessness or housing instability, single or teen parenthood, contact with child protection services). The ANRQ-R adheres to the recommendations of the SAFE START model, in terms of the core set of psychosocial variables that are assessed [14].

#### Key differences between the SAFE START and PIPA models of care

While the two psychosocial assessments are similar in content, PIPA differs from the SAFE START in a number of ways. The PIPA model: 1) considers in full all possible psychosocial risk factors identified across the booking visit and provides a *cumulative* measure of their impact by generating a total score on the ANRQ-R; 2) measures the *severity* of a number of risk factors (whereas SAFE START identifies only the presence or absence of these risk factors); 3) *auto-scores* the questionnaires, eliminating manual scoring errors arising in this context [25]; 4) includes additional structured questions to explore self-harm on the EPDS; 5) enables the midwife to document clinical concerns not elicited by the questionnaires, (e.g., the woman’s presentation at interview). This ‘Clinician Concerns’ option duly recognizes the importance of clinicians’ judgment and provides an avenue to refer a woman to the mental health midwife *even if* no risk is identified on the structured questionnaires.

### Setting

The site for the PIPA Project is the Royal Hospital for Women (RHW), a large teaching hospital in metropolitan Sydney, Australia, with approximately 4000 births per year [26].

During the first 12 months of the PIPA Project, women who attend the participating site for their first midwife-led routine antenatal visit will receive care as usual (SAFE START model), while women who attend for their antenatal booking-in visit in the last 12 months of the study will receive the alternative PIPA model of care. There will be an interval of approximately 12 months to allow for the implementation of a new state-wide administrative database (eMaternity), which will incorporate the PIPA model and one month of staff training for the PIPA model. During this time, women attending the hospital will continue to receive care as usual, though no study data will be collected (see Fig. 2).

### Time points for data collection

As shown in Fig. 2, data collected at two time points (Time 1 and Time 2) will inform the assessment of clinical- and cost-effectiveness outcomes, as well as women’s perceptions of the acceptability and perceived benefit of each model of care (Primary Aims). Data collected at these time points will also be used to examine the psychometric properties of a range of screening tools when used in pregnancy (Secondary Aim). Time 1 will be the initial psychosocial assessment (generally conducted at the first antenatal booking-in visit; approx.12 – 16 weeks gestation) and Time 2 will be approximately 2 weeks post-Time 1.

A range of data collection activities involving healthcare providers will comprise Time 3. Midwives will be approached to complete an emailed or paper-based survey (as per respondent preference) in the final six months of the data collection period for each model of care. Semi-structured key informant interviews with other healthcare professionals will also be conducted during this time. These data will be used to address the study’s clinical- and cost-effectiveness outcomes, as well as health care provider perceptions of the acceptability and perceived benefit of each model of care. Additional administrative and clinical data will be collected during periods of observation by the Research Officer throughout the allocation periods for each model of care, and focus groups will be held approximately 3 months after the introduction of the PIPA model.

### Consent processes for women attending the participating site

Permission to waive patient consent for the collection of Time 1 data has been granted by the governing Human Research Ethics Committee. For Time 2, women who express an interest in participating in this component of the study (indicated to the midwife at the time of the initial psychosocial assessment) will be subsequently contacted by research staff. At this time, women who agree to take part will give their informed consent.

### Participant eligibility and expected sample sizes

The estimated sample sizes for Time 1 and Time 2 is predicated on the number of births at the participating site (approximately 4000 women per annum), the proportion of these women who complete the initial psychosocial assessment (approx. 85%) and expected rates of participation at Time 2 (approx. 15-20% of all English-speaking women attending the site for their booking in visit).

*Time 1:* De-identified information relating to all women who attend the study site for their antenatal care will be included in the study database for Time 1, with an estimated total sample size of 7000 women completing a routine psychosocial assessment during the data collection periods for each model of care (3500 women per SAFE START and PIPA models of care).

*Time 2:* Only women who, at Time 1, respond ‘yes’ to an expression of interest (EoI) to be involved at Time 2 will be contacted by the Research Officer. Women who have not completed the routine psychosocial assessment during pregnancy, who are clients of the hospital’s Indigenous Midwifery service (due to differences in the models of care and service delivery) and/or who are unable to complete the Time 2 questionnaires in English will not be contacted to take part in Time 2. The anticipated total sample size for Time 2 is 1300 women (650 per model of care).

*Time 3:* Study participants will include up to 40 healthcare providers who are responsible for conducting the psychosocial assessment or providing emotional and mental health care for women who attend the participating site for antenatal care (representatives from psychiatry, social work, mental health nursing, midwifery, administration, hospital data management). All relevant staff will be invited to contribute data to Time 3 (via one or more of survey data, observational data, key informant interviews; focus groups); however, staff that provided care under only one of the models (i.e., SAFE START or PIPA) will be ineligible for focus group participation. The numbers of health care professionals anticipated to contribute data for each mode of data collection are: survey data (est. *n* = 25), observational data (est. *n* = 5–10), key informant interviews (est. *n* = 5–10) and focus groups (est. *n* = 8–16 individuals).

To enable economic assessments to be completed, data relating to time spent by midwives at booking, time spent by administrative staff collecting and distributing files for new bookings (approx. est. *n* = 30 files per staff member) will be recorded under both models of care. Data on the time spent discussing referrals to the social work department (*n* = 120), the mental health midwife (*n* = 120) and weekly MCD (*n* = 50) will also be recorded for each model of care. Information relating to the clinical ‘correctness’ of these referrals will also be collected: this will be determined by the consensus opinion of key senior clinicians at the weekly MCD (perinatal psychiatrist, mental health midwife, senior midwife, and senior social worker) or the weekly file review (‘triage’) meeting of the mental health midwife and senior social worker.

### Data sources, measures and procedures

#### Time 1: Initial psychosocial assessment conducted at the antenatal ‘booking in’ visit

The hospital data management system (ObstetriX and subsequently eMaternity) will be used to identify all women who attended the participating site during the data collection periods for the PIPA Project, and for whom a routine psychosocial assessment was completed. The Time 1 dataset will include: date of booking in visit and psychosocial assessment; gestation and maternal age at this time; maternal date of birth; estimated date of delivery; partner status; gravidity (including reason for pregnancy loss, where available); parity; country of birth; physical health conditions; type of conception; model of maternity care; EPDS: total and Q10 scores; EPDS: item level responses (SAFE START only); psychosocial questions: item level responses; psychosocial questions: total score (PIPA only); referral/s indicated as a result of the psychosocial assessment (yes/no); referral/s indicated as a result of the psychosocial assessment (specified; PIPA only); language; interpreter needed; financial class (public, private or overseas patient); and Time 2 Expression of Interest response (yes/no). Time 1 data will be extracted by the data manager at the participating site at approximately weekly intervals, for all women allocated to the SAFE START and PIPA models of care.

#### Time 2: Two weeks post-time 1

Women who consent to participation at Time 2 will complete the EPDS [6], the ‘Whooley’ questions [19] followed by a third ‘help’ question [20], the Generalized Anxiety Disorder Scale [GAD-7] [21], the Matthey Generic Mood Questionnaire (MGMQ) [22], an ‘interval’ question to assess perceived change in emotional wellbeing at Time 2 [27] and the Mini International Neuropsychiatric Interview v6.0 [M.I.N.I.; mood and anxiety disorder modules only] [23]. Women will also complete a short survey covering the acceptability of the psychosocial questions and assessment process.

‘Key Survey’™ will be used as the platform for data collection for all Time 2 measures *except* the M.I.N.I., which will be collected using infrastructure provided by Medical Outcomes Systems (to whom the online M.I.N.I. is copyrighted). Alternatively, these questionnaires will be administered by phone by the Research Officer verbatim, with participant responses captured in the project-specific ‘Key Survey’ and MINI instruments.

#### Time 3: various data collection points during the model of care allocation periods

*Survey for health professionals:* Study-specific survey questions will address a range of issues related to the experience of healthcare professionals (in particular, midwives) in providing psychosocial care to women during pregnancy, including: level of comfort in asking the psychosocial questions; level of confidence in discussing responses to the psychosocial assessment and (if required) referral and support options; time spent administering the initial psychosocial assessment; time spent initiating any referrals; ease of use of computer-based assessment tool; and utility of referral advice and the psychosocial summary report (listing key issues and referral prompts arising from the psychosocial assessment), for each model of care. ‘Key Survey’™ will be used as the platform for data collection for the healthcare professional feedback questionnaire. Paper versions of the questionnaires will be provided to healthcare professionals who do not have internet access, or who would prefer to complete the measures in hardcopy.

*Observational data and key informant interviews:* The following information will be collected for each model of care: time spent by clinical and administrative staff on administrative tasks directly related to psychosocial care, including collecting and distributing files for new bookings where a psychosocial referral was made; time spent ‘triaging’ and allocating these files as appropriate; time spent on case discussion at the MCD for new bookings; and other relevant administrative tasks.

As part of the Time 3 data collection, the Research Officer will also attend the weekly psychosocial ‘triage’ and MCD meetings to collate referral pathway data for women in each model of care. This will include information on the number and proportion of women for whom a referral was made from their initial psychosocial assessment, and the number and proportion of women considered ‘correct’ and ‘incorrect’ referrals. The Research Officer, in consultation with the relevant on-site departments and clinicians, will collate this information for both models of care. This process will also be facilitated by the Research Officer observing/shadowing the day-day activities of key staff.

*Focus groups:* Health care professionals providing psychosocial care for pregnant women at the study site will be invited to participate in a study-specific focus group. These may include midwives, social workers, mental health midwives and psychiatrists. The focus groups will explore participants’ experiences of providing the alternative models of psychosocial care, including (but not limited to) time and ease of administration, understanding of risk levels (including nomenclature) and perception of care quality for the woman. A question guide will be used to facilitate discussion and will be informed by key themes from existing literature, consultation with experts and feedback captured in the Time 3 surveys. Each focus group will be approximately 60–90 min in duration. Discussion will be recorded with the permission of participants using digital audio recorders and will be transcribed verbatim.

#### Resource consumption and unit resource cost estimates

The economic evaluations will be informed by survey data, observational data, key informant interviews and focus groups during the trial period and will be completed at Time 3. Bottom up costing methods based on the principle of opportunity costs forgone will be employed to capture the direct costs of identifying ‘correct’ and ‘incorrect’ referrals cases. The Research Officer will time each activity associated with completing psychosocial assessment and administrative tasks associated with the initiation of referrals to various site-based support services (see Box). This will allow the allocation of costs for each activity for inclusion in the cost-effectiveness analysis. The salary costs applied for each unit of activity will be taken from NSW Health Service Awards. Overhead costs will not be applied because they are equivalent between the two models of care. Because the PIPA Project has been designed to primarily inform decision making around effectiveness, efficiency and acceptability of a psychosocial screening program from the perspective of the maternity sectors, indirect patient costs associated with travel etc will not be included, nor will the costs associated with downstream, clinical care or interventions and the broader burden associate with mental illness. Costs will be presented in constant Australian dollars (AUD) (Table 2).

### Statistical analysis

#### Clinical effectiveness, acceptability and perceived benefit analysis

Effectiveness outcomes (Primary Aim 1), including the proportion of women identified at particular psychosocial risk levels (as defined in the SAFE START and PIPA models of care) and proportion of women considered to be ‘correct’ or ‘incorrect’ referrals in each care model will be analysed using chi-square and independent-samples *t*-tests (or Mann Whitney U tests, as appropriate). This approach will also be used to examine the acceptability and perceived benefit of the SAFE START and PIPA models (Primary Aim 3). Initial checking of statistical assumptions (normality, multicollinearity, examination of outliers) will be undertaken. Chi-square analysis of subgroups of women based on risk status may also be explored. Clinically significant differences between the two models will be based upon obtaining at least medium effect sizes or absolute percentage differences of at least 15%.

#### Cost-effectiveness analysis

The primary outcome variable for the cost-effectiveness analysis (Primary Aim 2) will be the proportion of ‘correct’ referrals initiated and ‘incorrect’ referrals averted. The incremental costs and effects of SAFE START compared to PIPA will be expressed as the incremental cost-effectiveness ratio (ICER). The ICER combines the net changes in effects with the net change in costs between two strategies providing a relative estimate of the cost-effectiveness of the PIPA program relative to the SAFE START program. The ICER per ‘correct’ referral made and per ‘incorrect’ referral averted will be estimated using mean costs and effects and represented with 95% confidence intervals for the ICERs using non-parametric bootstrapping techniques. One-way and multivariate probability sensitivity analysis using Monte Carlo simulations across the cost and effect distributions will be undertaken to investigate uncertainty around the ICERs. Based on number of women screened and proportion of ‘correct’ and ‘incorrect’ referrals identified, the economic evaluation will allow a budget impact analysis to be undertaken to determine the costs of running SAFE START and PIPA at other maternity hospitals.

#### Focus groups (qualitative data)

The qualitative focus group data will be analysed by two members of the research team with qualitative expertise using inductive thematic content analysis consistent with an interpretive descriptive approach. Data will be entered into NVivo. Transcribed data will be coded first by using an open coding process to organise the data by identifying broad thematic patterns [28]. The data will then be read and re-read to enable classification into categories and specific themes [28, 29].

#### Psychometric properties of screening tools

To examine the psychometric properties of the EPDS, ‘Whooley’ questions, GAD-2 and MGMQ when used during pregnancy (Secondary Aim), we will conduct a receiver operator curve analysis to calculate the area under the curve and sensitivity, specificity, positive and negative predictive values and positive and negative likelihood ratios negative likelihood ratios of each measure, using the M.I.N.I. as the gold standard (mood and anxiety disorder modules only). Consistent with other studies of this type a value of 0.5 will be added if a 2x2 cell contains zero, in order to calculate the likelihood ratios and associated confidence intervals. In addition the various screening measures will be compared with each other, using each measure’s screen-positive threshold (where these thresholds are available).

### Study status

This study is ongoing. Data collection for the SAFE START model of care (care as usual) is complete. Data collection for the PIPA model of care (alternative model) is scheduled to commence in the first half of 2017.

### Discussion

The value of an integrated approach to depression screening in facilitating the connection between assessment processes and care pathways has been demonstrated in the general population literature [30–32] and is gaining increased attention in the perinatal context [16, 33–35]. However, existing perinatal studies, like general population studies, have been limited to depression screening alone, rather than more comprehensive psychosocial assessment (which is inclusive of, but not limited to, depression screening). This is the first study of its kind to incorporate an evaluation of how broader components of integrated care, including electronic clinician decision-making aids and referral processes, may influence the outcomes of a program.

The PIPA Project will contribute substantially to the literature by providing evidence for the value of structured psychosocial assessment tools and electronic decision making algorithms in a large scale study. The present study will provide crucial insight into the most cost-effective use of health resources and information about potential sources of cost reduction (via improved resource efficiency in the public maternity sector). Through its secondary aim, the PIPA Project will also respond to calls made in the Australian and UK Clinical Practice Guidelines for further research evaluating the psychometric properties of screening tools for depression and anxiety in pregnant women.

## Abbreviations

ANRQ-R: Antenatal risk questionnaire-revised
EPDS: Edinburgh perinatal depression scale
GAD-2: Generalized anxiety disorder scale-2 items
M.I.N.I.: Mini international neuropsychiatric interview (v6.0)
MCD: Multidisciplinary case discussion meeting
MGMQ: Matthey generic mood questionnaire
PIPA: Perinatal integrated psychosocial assessment
